# The Impact of Suicidality-Related Internet Use: A Prospective Large Cohort Study with Young and Middle-Aged Internet Users

**DOI:** 10.1371/journal.pone.0094841

**Published:** 2014-04-16

**Authors:** Hajime Sueki, Naohiro Yonemoto, Tadashi Takeshima, Masatoshi Inagaki

**Affiliations:** 1 Department of Psychology and Education, Faculty of Human Sciences, Wako University, Machida, Tokyo, Japan; 2 Department of Epidemiology and Biostatistics, Translational Medical Center, National Center of Neurology and Psychiatry, Kodaira, Tokyo, Japan; 3 Center for Suicide Prevention, National Institute of Mental Health, National Center of Neurology and Psychiatry, Kodaira, Tokyo, Japan; 4 Department of Neuropsychiatry, Okayama University Hospital, Okayama, Japan; National Institute of Mental Health, Japan

## Abstract

**Background:**

There has been no study that has allowed clear conclusions about the impact of suicide-related or mental health consultation-related internet use.

**Aim:**

To investigate the impacts of suicide-related or mental health consultation-related internet use.

**Methods:**

We conducted prospective observational longitudinal study with data collection at baseline screening (T0), 1 week after T0 (T1) and 7 weeks after T0 (T2). Participants with a stratified random sampling from 744,806 internet users were 20–49 years of age who employed the internet for suicide-related or mental health consultation-related reasons and internet users who did not. The main outcome was suicidal ideation. Secondary outcome measures comprised hopelessness, depression/anxiety, and loneliness.

**Results:**

The internet users who had employed the internet for suicide-related or mental health consultation-related reasons at T0 (*n* = 2813), compared with those who had not (*n* = 2682), showed a significant increase in suicidal ideation (*β* = 0.38, 95%CI: 0.20–0.55) and depression/anxiety (*β* = 0.37, 95%CI: 0.12–0.61) from T1 to T2. Those who disclosed their own suicidal ideation and browsed for information about suicide methods on the web showed increased suicidal ideation (*β* = 0.55, 95%CI: 0.23–0.88; *β* = 0.45, 95% CI: 0.26–0.63, respectively). Although mental health consultation with an anonymous other online did not increase suicidal ideation, increased depression/anxiety was observed (*β* = 0.34, 95%CI: −0.03–0.71).

**Conclusions:**

An increased suicidal ideation was observed in the young and middle-aged who employed the internet for suicide-related or mental health consultation-related reasons. Mental health consultation via the internet was not useful, but those who did so showed worsened depression/anxiety.

## Introduction

Suicide is a critical global issue with a global mortality rate of 16 per 100,000 [1]. More specifically, suicide rates in young people have risen [2]. Suicide is among the top 20 leading causes of death globally for all ages and among the three leading causes of death among those aged 15–44 years in some countries [1]. Previous studies have shown that many suicidal people do not seek help and treatment [3]. Reasons for not seeking help have been reported as stigma and temporal/financial constraints [4] [5]. As a consequence, the internet may be useful for providing information and help for those who are suicidal, especially young and middle-aged persons, because it is anonymous, low cost, and easy to use [6]. Previous studies have discussed how the internet has both suicide-preventive and suicide-inducing effects [7]–[10]. Information about suicide methods was possibly categorized by expert consensus as pro-suicidal [11] [12], and provision of consulting about mental health (e.g., email-based crisis intervention) as anti-suicidal [13]–[15].

However, there has been no large-sized prospective cohort study or randomized controlled trial that has allowed any causal conclusions about the impact of suicidality-related internet use. Cross-sectional studies have reported an association of internet usage with suicidal ideation [16] [17]. In addition, a previous study reported the association of suicide information availability on the internet with suicide methods used among those who died by suicide, based on inquests [18]. Previous studies did not reach a clear conclusion about the effects of suicidality-related internet use [19].

Therefore, we investigated whether suicidality-related internet use (disclosing one's suicidal ideation, mental health consultation, and browsing for information about suicide methods) is related to changes in suicidal ideation and other mental health status items related to suicide (hopelessness, depression/anxiety, and loneliness) in a prospective observational longitudinal study. The hypothesis tested was that suicidality-related internet use affects the users' suicidal ideation and the other mental health scores.

## Methods

### Participants

A survey was made of internet users who were between 20 and 49 years of age with Japanese literacy. Minors were excluded from our research target groups for ethical reasons. We did not include those in their 50 s or older because of the finding that this group is seldom included in surveys of suicidality-related internet users [16].

At the screening survey, we excluded individuals who had planned or attempted suicide within the past month to avoid any encouragement of suicidal behaviours. In addition, participants with incomplete or untrustworthy answers (e.g., answers including incomprehensible character strings in open-response questions) were also excluded because these answers would not be reliable.

### Study design

**Figure 1:**
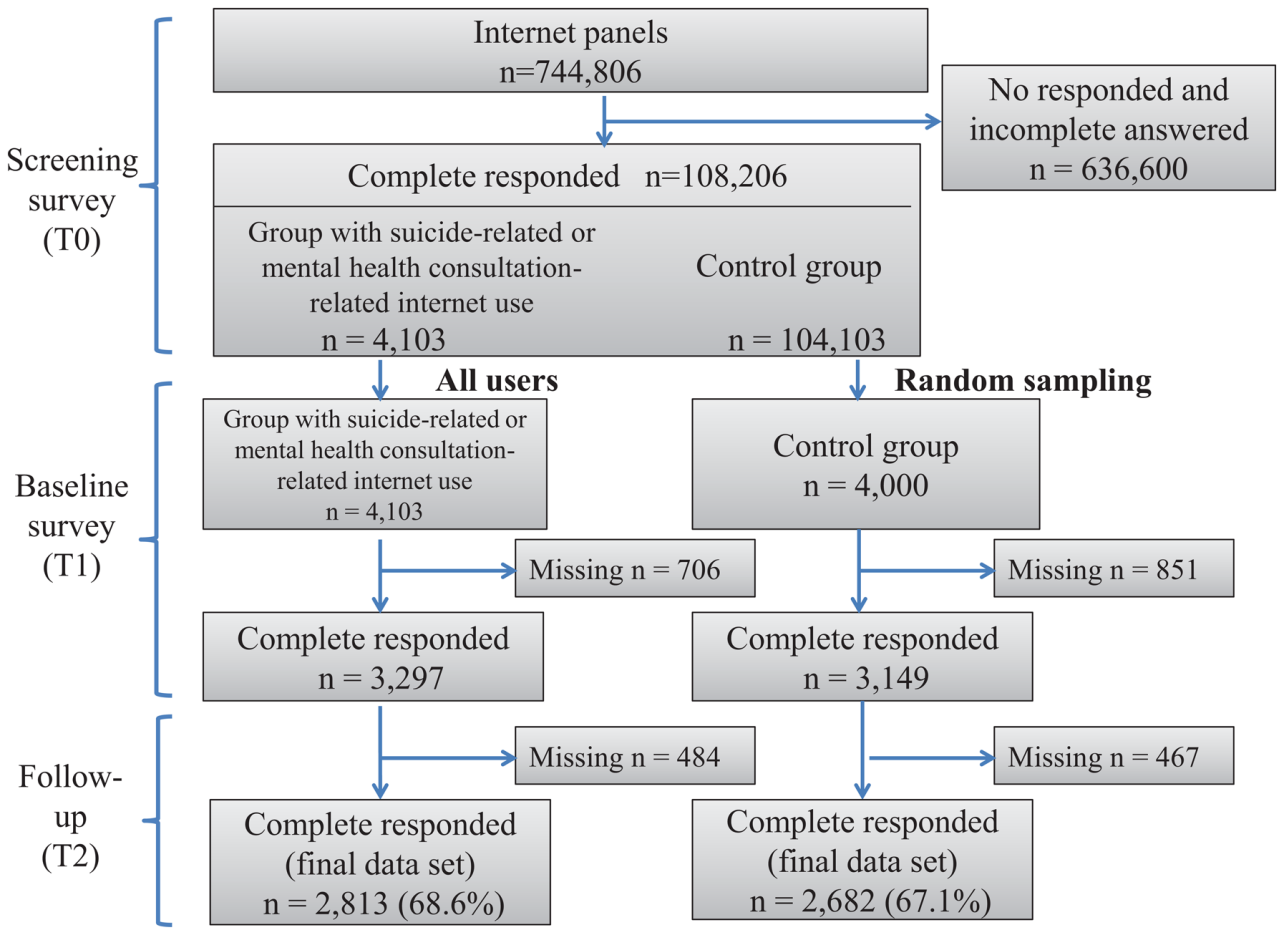
Flow chart of the study. This shows the sampling process of this study.

The study was a prospective observational longitudinal study. The baseline screening survey (T0 survey) and two waves (T1 [1 week after T0] and T2 [7 weeks after T0]) of follow-up surveys were conducted with members of comprehensive internet survey panels through a major Japanese internet survey company (Cross Marketing Inc., Tokyo, Japan) (see Figure 1). The T0 survey was based on a target population of those from 20 to 49 years of age distributed according to the demographics of the census data of 2005 in Japan [20], with stratified random sampling of 744,806 internet panel participants (about 20,000 internet panel participants in each of the groups of ages 20–29, 30–39, and 40–49 years). The stratified variables were age, sex and geographic region of residence. The sample size chosen was based on an expected response rate of 10% or less [21]. This sample size would have over 90% power to detect an expected regression coefficient of 0.3 in the multivariate regression analysis.

At the T0 survey, we asked the following four questions and defined the participants who endorsed any of them as the group with suicide-related or mental health consultation-related internet use: “Q1: Over the past month, have you disclosed your wish to commit a suicide to an anonymous other on the Internet?”, “Q2: Over the past month, have you consulted with an anonymous other about your mental health on the Internet?”, “Q3: Over the past month, have you browsed for information concerning suicide methods on the Internet?”, and “Q4: Have you ever disclosed your wish to commit suicide to an anonymous other on the Internet?”.

In the T1 survey, a questionnaire was given to the participants who answered “Yes” to any of the questions Q1, 2, 3 and 4 (the group with suicide-related or mental health consultation-related internet use) at the T0 survey. Also, a random sample was taken of the participants who answered “No” to all four questions at the T0 survey and defined as the control group. In the T2 survey, a questionnaire was distributed to all participants who completed the T1 survey.

### Ethical considerations

The study was approved by the ethical review board at The University of Tokyo, Japan (Registration number: 10–24, http://www.u-tokyo.ac.jp/ja/administration/lifescience/), and complied with the ethical guidelines for epidemiology research by the Ministry of Health, Labour and Welfare, Japan. We briefed survey participants on the possibility that viewing or responding to the questionnaire might lead to a mood change before they consented to participate in the study, and then obtained informed consent from participants online. In addition, links to websites containing professional support resources were shown to participants occasionally during the time they were completing the questionnaire.

### Measurements

All participants answered a self-administered questionnaire on the internet containing questions about suicide-related or mental health consultation-related internet use, suicidal ideation, hopelessness, depression/anxiety, loneliness, and coping with stress (see Table 1).

**Table 1:** List of the survey items at T0, T1, and T2.

	Item	Reference
**Screening Survey (T0)**	**Characteristics of participants**	
	Sex	
	Age	
	Educational background	
	Marital status	
	Household income	
	Drinking alcohol	
	Smoking	
	Hospital visit	
	Time spent online per day	
	**Suicide-related Internet use**	(Sueki, 2013)
	Disclosing one's suicidal ideation	
	Mental health consultation with anonymous other	
	Browsing for information about suicide methods	
**Baseline Survey (T1)**	**Stress coping**	(Ozeki, 1993)
	**Mental state**	
	Suicidal ideation	(Beck et al., 1979)
	Hopelessness	(Beck et al., 1974)
	Depression/Anxiety tendency	(Kessler et al., 2002)
	Loneliness	(Ochiai, 1983)
	**Lifetime suicidal behaviours**	
	Deliberate self harm	
	Thoughts of death	
	Thoughts of suicide for revenge	
	Thoughts of suicide as the only way of solving the problem	
	Suicide plan	
**Follow up (T2)**	**Mental state**	
	Suicidal ideation	(Beck et al., 1979)
	Hopelessness	(Beck et al., 1974)
	Depression/Anxiety tendency	(Kessler et al., 2002)
	Loneliness	(Ochiai, 1983)

At the T0 survey, Yes-No questions were asked about experience with suicide-related or mental health consultation-related internet use: disclosing one's suicidal ideation within a month (Q1), mental health consultation within a month (Q2), browsing for information about suicide methods within a month (Q3), and lifetime experience of disclosing one's suicidal ideation (Q4). All items were made by reference to our previous related study about suicide-related internet use [22].

The Scale for Suicide Ideation designed by Beck and his colleagues [23] was used to measure suicidal ideation at T1 and T2. This scale is a 19-item clinical research instrument designed to quantify and assess suicidal intention. A score of 0–2 is given to each response, with higher points representing increased levels of suicidal ideation. It should be noted that the original scale was designed to be given by a trained administrator, while the version used in this study (Japanese version) is a self-rating scale with questions modified in light of the Japanese social-cultural environment (e.g., the item about gun-related suicide was eliminated). The Japanese version of this scale, consisting of 13 items (score range  = 0–26), was confirmed for reliability (Cronbach's *α* = 0.85) and validity through a survey with 344 college students [24].

The Beck Hopelessness Scale was used to measure hopelessness at T1 and T2 [25].This scale is a 20-item true-false self-report instrument that assesses the degree to which a person holds negative expectations about the future. The items are summed to obtain a total hopelessness score (range  = 0–20). The Japanese version of this scale showed a high degree of reliability (Kuder–Richardson Formula 20 = 0.86) and validity through a survey with 160 college students [26].

The K6 (six items) was used to measure depression/anxiety tendency at T1 and T2 [27]. The K6 is an abridged version of the Kessler Psychological Distress Scale (K10), a scale based on the item response theory for effectively detecting mental disorders. For each item, responses were rated on a 5-point scale ranging from 1 point for “Not at all” to 5 points for “Always.” Scores could vary from 6 to 30. The Japanese version was developed and has been shown to be equal in screening performance to the original [28].

Loneliness was measured by Ochiai's Loneliness Scale (nine items) at T1 and T2 [29]. Responses to questions (e.g., “I don't think there is anyone sympathetic to whom I can turn to for advice”) were rated using a 5-point scale ranging from 5 points for “Yes” to 0 points for “No.” The validity of this scale has been confirmed on the basis of its association with the Revised UCLA Loneliness Scale [30]. In addition, it was confirmed to have a high reliability (*r*: correlation coefficient, 0.66<*r*<0.83) according to the test-retest method at intervals of 1 month and 6 months.

The Coping Scale (14-item) was used to measure stress coping at T1 [31]. This scale was designed to measure an individual's coping ability with the most important stressor that he/she is experiencing at a given moment in the simplest manner possible. It consists of three subscales: five items for problem-focused type coping (item example: making an effort to change the present situation) and three items for emotion-focused type coping (item example: encouraging oneself), both as positive coping, as well as six items for avoidance/escape type coping, as passive coping (item example: trying not to think of the future). A question was asked for each item, and the response to the question was rated by using a 3-point scale from 0 points for “I don't do that at all” to 3 points for “I always do that.” The reliability and validity of this scale was confirmed through a survey of over 599 college students. Cronbach's *α* values for the problem-focused, emotion-focused, and avoidance/escape subscales were 0.75, 0.76, and 0.72, respectively.

### Statistical analyses

We analysed the data for the impact of suicide-related or mental health consultation-related internet use from those who responded and gave complete answers in the surveys. The descriptive statistics show the differences in characteristics of participants between the group with suicide-related or mental health consultation-related internet use and the control group. We checked the impacts of non-responders and missing cases for the analysis. In a comparison of the characteristics between participants with and without suicide-related or mental health consultation-related internet use experience, the t-test was employed for continuous data and the chi-square test was used for binary data.

For our primary analyses to examine the relationships of suicide-related or mental health consultation-related internet use with users' suicidal ideation and the other mental health scores, multivariate regression models were performed. The aim was to investigate whether those with suicide-related or mental health consultation-related internet usage showed changes in scores for suicidal ideation, hopelessness, depression/anxiety, and loneliness from T1 to T2, adjusting for the participants' characteristics, coping skills, and other mental health scores at T1. This is because these mental health scores, demographics and coping skills have been reported previously to have an influence on both suicidal ideation and internet usage [25] [32].

In this analysis, there were two types of independent variables of suicide-related or mental health consultation-related internet use. First, we analysed the models where the independent variable was whether or not participants endorsed any of the four questions about suicide-related or mental health consultation-related internet use. Second, the models for each of the four types of suicide-related or mental health consultation-related internet use (disclosing one's suicidal ideation within a month, mental health consultation, browsing for information about suicide methods, and disclosing one's suicidal ideation up until a month ago) were analysed separately with each suicide-related or mental health consultation-related internet use as the independent variable in the model. We examined three versions of the model: Model 1 (minimally adjusted) controlling for T1 mental health scores (suicidal ideation, hopelessness, depression/anxiety, and loneliness); Model 2 controlling for T1 mental health scores and the participants' characteristics (educational background, marital status, household income, drinking alcohol, smoking, psychiatric hospital visit, and time spent online per day); and Model 3 (fully adjusted) controlling for T1 mental health scores, the participants' characteristics, and coping skills (problem-focused, emotion-focused and avoidance/escape). In these three models, we added potential confounders in the order corresponding to the strength of the relationship to suicidal ideation, because robustness of the results needed to be checked.

There were strong observed confounders between the two groups. Therefore, we adjusted for these confounders. The methods for control of confounding are of various types, including restriction, matching, stratification and regression modeling. Multivariate regression modelling is more flexible and can fully adjust strong observed confounders for targeted populations with high validity. Other methods like matching have some limitations from a modern epidemiological perspective. In the other methods, the numbers of adjustment factors are limited. Second, these methods carry risks of over or under adjustments. Third, adjusted results using these methods would not be representative of the targeted population. Only regression modeling can overcome these limitations.

In the regression model, we selected confounding factors in the model on the basis of prior knowledge of risk factors, because the confounders could not be identified in the observed data [33]. However, the knowledge from previous studies was limited. Therefore, we performed three models with potential confounders to check the sensitivity of the confounders, step-by step. Model 3 (the full model) allowed adjustment for all observed confounders.

Regression coefficients (*β*) and their 95% confidence intervals (CI) were calculated in the models. The p-values presented are for two-tailed tests. The analysis was performed using SPSS software (SPSS 19.0 for Windows; SPSS Inc., Chicago, IL). The present study is in accordance with the STrengthening the Reporting of OBservational studies in Epidemiology (STROBE) statement.

## Results

### Characteristics of the participants

The primary number of analysed participants was 5495: 43.3% were women and the mean age was 35.5 years (standard deviation  = 7.8, range  = 20–49); 2813 participants were the group with suicide-related or mental health consultation-related internet use and 2682 were the control group (see Figure 1). No participants were excluded because of their suicidality. There were no missing values for any variable in the final data set.

Characteristics of the participants are shown in Table 2. Significant differences in the proportions of age, marital status, household income, drinking alcohol, smoking, and hospital visit were found between the group with suicide-related or mental health consultation-related internet use and the control group at the T0 survey. There were no differences of gender and education. Compared with the control group at T0, the group with suicide-related or mental health consultation-related internet use were likely to have lower scores for coping with stress and higher scores for mental health problems at T1. The proportion of lifetime suicidal behaviours of the group with suicide-related or mental health consultation-related internet use was significantly greater than that of the control group at T1.

**Table 2:** Comparison between the group with suicide-related or mental health consultation-related internet use and the control group who completed T2 survey.

	Group with suicide-related or mental health consultation-related internet use (*n* = 2813)	Control group (*n* = 2682)	Difference	*p*
**Characteristics of participants**				
Male: n (%)	1587 (56.4)	1529 (57.0)	−0.6	0.663
Age: mean (s.d.)	34.1 (7.9)	36.9 (7.5)	−2.8	<0.001
Educational background, junior high and high school graduate: n (%)	825 (29.3)	729 (27.2)	2.1	0.082
Marital status, not married: n (%)	1724 (61.3)	1140 (42.5)	18.8	<0.001
Household income less than 4 million yen (USD $40,000) a year: n (%)	1092 (38.8)	731 (27.3)	11.5	<0.001
Drinking alcohol more than once a week: n (%)	1315(48.5)	1498(53.8)	−5.3	<0.001
Smoking more than once a day: n (%)	873(31.0)	59.3(21.7)	9.3	<0.001
Hospital visit (Present, Total): (%)	1248 (44.4)	693 (25.8)	18.6	<0.001
Hospital visit (Present, Psychiatry and/or Psychosomatic Internal): (%)	658 (23.4)	119 (4.4)	19.0	<0.001
Time spent online per day: mean (s.d.)	3.5 (1.4)	2.7 (1.6)	0.8	<0.001
**Suicide-related Internet use**				
Disclosing one's suicidal ideation (up until a month ago): n (%)	1292(45.9)	0(0)	45.9	<0.001
Disclosing one's suicidal ideation (within a month): n (%)	331(11.8)	0(0)	11.8	<0.001
Mental health consultation with anonymous other (within a month): n (%)	497(17.7)	0(0)	17.7	<0.001
Browsing for information about suicide methods (within a month): n (%)	1242(44.2)	0(0)	44.2	<0.001
**Stress coping scores: mean (s.d.)**				
Problem-focused	6.0 (3.2)	6.5 (3.1)	−0.5	<0.001
Emotion-focused	3.5 (2.4)	4.5 (2.3)	−1.0	<0.001
Avoidance/escape	8.5 (3.7)	9.2 (3.7)	−0.7	<0.001
**T1 Mental state scores: mean (s.d.)**				
Suicidal ideation	8.5 (5.7)	2.4 (3.3)	6.1	<0.001
Hopelessness	13.0 (5.0)	8.4 (5.0)	4.6	<0.001
Depression/Anxiety tendency	17.0 (5.9)	11.0 (4.6)	6.0	<0.001
Loneliness	1.1 (8.7)	−5.0 (8.1)	6.1	<0.001
**T2 Mental state scores: mean (s.d.)**				
Suicidal ideation	8.0 (5.7)	2.4 (3.3)	5.6	<0.001
Hopelessness	13.0 (4.8)	8.6 (4.8)	4.4	<0.001
Depression/Anxiety tendency	16.7 (6.1)	11.1 (4.8)	5.6	<0.001
Loneliness	0.9 (8.8)	−4.9 (8.1)	5.8	<0.001
**T1 Lifetime suicidal behaviours: number (%)**				
Deliberate self harm	874 (31.1)	209 (7.8)	23.3	<0.001
Thoughts of death	2091 (74.3)	764 (28.5)	45.8	<0.001
Thoughts of suicide for revenge	972 (34.6)	247 (9.2)	25.4	<0.001
Thoughts of suicide as the only wayof solving the problem	1691 (60.1)	463 (17.3)	42.8	<0.001
Suicide plan	1246 (44.3)	228 (8.5)	35.8	<0.001

The t-test was employed for continuous data, and the chi-square test was used for categorical data.

Drinking alcohol, smoking and hospital visit had some differences between dropouts and completed responders in the group with suicide-related or mental health consultation-related internet use. However, in the control group, there were no differences in drinking alcohol, smoking and hospital visit.

### Relationships between suicide-related or mental health consultation-related internet use and mental health score changes from T1 to T2

As seen in Table 3, the regression models show the relationships between suicide-related or mental health consultation-related internet use and changes of suicidal ideation, hopelessness, depression/anxiety, and loneliness from T1 to T2. Statistically significant positive coefficients were found for suicidal ideation (*β* = 0.38 [95%CI: 0.20 to 0.55]) and depression/anxiety (*β* = 0.37 [95%CI: 0.12 to 0.61]). The scores for hopelessness and loneliness did not show any effects of suicide-related or mental health consultation-related internet use.

**Table 3:** Relationships between suicide-related or mental health consultation-related internet use and changes in mental health scores between T1 and T2.

	Suicidal ideation (T2-T1)			Hopelessness (T2-T1)			Depression/Anxiety (T2-T1)			Loneliness (T2-T1)		
	*β*	95%CI	*P*	*β*	95%CI	*P*	*β*	95%CI	*P*	*β*	95%CI	*P*
Model 1	**0.40**	**0.23–0.57**	**<0.001**	0.05	−0.13–0.23	0.590	**0.56**	**0.31–0.80**	**<0.001**	−0.04	−0.39–0.32	0.835
Model 2	**0.36**	**0.19–0.53**	**<0.001**	0.07	−0.11–0.26	0.423	**0.38**	**0.13–0.63**	**0.003**	0.00	−0.36–0.36	0.994
Model 3	**0.38**	**0.20–0.55**	**<0.001**	0.11	−0.07–0.29	0.242	**0.37**	**0.12–0.61**	**0.004**	0.04	−0.32–0.41	0.815

CI: Confidence interval.

Bold type indicates significance (P<0.05).

Model 1 (minimally adjusted): Controlled variables were T1 mental health scores (suicidal ideation, hopelessness, depression/anxiety, and loneliness).

Model 2: Controlled variables were T1 mental health scores and characteristics of participants.

Model 3 (fully adjusted): Controlled variables were T1 mental health scores, characteristics of participants, and coping style scores.

Table 4 reports the regression coefficients of each suicide-related or mental health consultation-related internet use. Disclosing one's suicidal ideation up until a month ago (*β* = 0.37, 95%CI: 0.17 to 0.57), disclosing one's suicidal ideation within the last month (*β* = 0.55, 95%CI: 0.23 to 0.88), and browsing for information about suicide methods within a month (*β* = 0.45, 95%CI: 0.26 to 0.63) significantly increased suicidal ideation from T1 to T2. Internet mental health consultation with an anonymous other within the last month did not increase suicidal ideation. We obtained a similar result in the other three models for suicidal ideation.

**Table 4:** Relationships between each type of suicide-related or mental health consultation-related internet use and suicidal ideation, hopelessness, depression/anxiety, and loneliness.

		Suicidal ideation (T2-T1)			Hopelessness (T2-T1)			Depression/Anxiety (T2-T1)			Loneliness (T2-T1)		
		*β*	95%CI	*P*	*β*	95%CI	*P*	*β*	95%CI	*P*	*β*	95%CI	*P*
Model 1	Disclosing one's suicidal ideation (up until a month ago)	**0.41**	**0.21–0.60**	**<0.001**	0.07	−0.13–0.28	0.480	**0.51**	**0.23–0.79**	**<0.001**	−0.13	−0.54–0.23	0.534
	Disclosing one's suicidal ideation (within a month)	**0.62**	**0.30–0.94**	**<0.001**	−0.24	−0.58–0.10	0.163	0.31	−0.15–0.78	0.186	0.07	−0.61–0.75	0.834
	Mental health consultation with anonymous other (within a month)	−0.10	−0.35–0.16	0.447	−0.09	−0.36–0.18	0.516	**0.54**	**0.17–0.91**	**0.004**	−0.13	−0.67–0.40	0.629
	Browsing for information about suicide methods (within a month)	**0.41**	**0.23–0.60**	**<0.001**	0.02	−0.17–0.21	0.835	**0.29**	**0.02–0.55**	**0.033**	0.23	−0.16–0.62	0.247
Model 2	Disclosing one's suicidal ideation (up until a month ago)	**0.36**	**0.16–0.55**	**<0.001**	0.06	−0.14–0.27	0.551	**0.31**	**0.02–0.59**	**0.033**	−0.04	−0.46–0.37	0.836
	Disclosing one's suicidal ideation (within a month)	**0.55**	**0.23–0.88**	**0.001**	−0.20	−0.55–0.14	0.244	0.10	−0.36–0.57	0.668	0.24	−0.44–0.93	0.484
	Mental health consultation with anonymous other (within a month)	−0.16	−0.41–0.10	0.230	−0.04	−0.31–0.23	0.785	0.36	−0.01–0.73	0.059	0.05	−0.49–0.59	0.850
	Browsing for information about suicide methods (within a month)	**0.43**	**0.24–0.62**	**<0.001**	0.05	−0.14–0.25	0.601	0.26	−0.01–0.53	0.056	0.13	−0.26–0.52	0.519
Model 3	Disclosing one's suicidal ideation (up until a month ago)	**0.37**	**0.17–0.57**	**<0.001**	0.08	−0.12–0.29	0.432	**0.30**	**0.02–0.58**	**0.038**	−0.02	−0.43–0.40	0.936
	Disclosing one's suicidal ideation (within a month)	**0.55**	**0.23–0.88**	**0.001**	−0.18	−0.52–0.16	0.297	0.10	−0.36–0.57	0.662	0.26	−0.42–0.94	0.459
	Mental health consultation with anonymous other (within a month)	−0.15	−0.41–0.11	0.248	−0.01	−0.28–0.26	0.959	0.34	−0.03–0.71	0.069	0.09	−0.45–0.63	0.738
	Browsing for information about suicide methods (within a month)	**0.45**	**0.26–0.63**	**<0.001**	0.08	−0.12–0.27	0.455	0.26	−0.01–0.53	0.062	0.16	−0.24–0.55	0.433

CI: Confidence interval.

Bold type indicates significance (P<0.05).

Model 1 (minimally adjusted): Controlled variables were T1 mental health scores (suicidal ideation, hopelessness, depression/anxiety, and loneliness).

Model 2: Controlled variables were T1 mental health scores and characteristics of participants.

Model 3 (fully adjusted): Controlled variables were T1 mental health scores, characteristics of participants, and coping style scores.

Those who disclosed their suicidal ideation up until a month ago showed increased depression/anxiety (*β* = 0.30, 95%CI: 0.02 to 0.58) from T1 to T2. Those with mental health consultation within a month (*β* = 0.34, 95%CI: −0.03 to 0.71) and browsing for information about suicide methods within a month (*β* = 0.26, 95%CI: −0.01 to 0.53) showed increased depression/anxiety also. The scores for hopelessness and loneliness did not show any effects of suicide-related or mental health consultation-related internet use.

We described the differences between primary analysed cases and non-responders and missing cases. There were few differences in background of the participants. However, the main results of our analysis were still robust. (data was not shown).

## Discussion

### Principal findings

Disclosing one's suicidal ideation and browsing for information about suicide methods increased suicidal ideation. Mental health consultation with anonymous others by internet users did not increase suicidal ideation but increased depression/anxiety. Our large-sized prospective longitudinal study confirmed the effects of suicide-related internet use on suicidality. Those with suicide-related or mental health consultation-related internet use showed increased suicidal ideation and depression/anxiety, and the three types of suicide-related internet use were each independently related to these same changes in mental health scores. As in previous studies [11] [22], our results extended and strengthened the causal link between browsing for information about suicide methods online and increased suicidal ideation. Those who disclosed suicidal ideation, which was considered to be suicide-preventive in some previous studies [14] [16], showed increased suicidal ideation. Those who had mental health consultation with an anonymous other showed no effect on suicidal ideation, but had increased depression/anxiety. We showed a negative effect of disclosing one's suicidal ideation online, which dovetailed with the indication that there was the potential harm of a downward depressive spiral in online anonymous communities consisting of users with depressive tendencies [34].

The effect size of suicide-related or mental health consultation-related internet use might be interpreted as relatively small. Each suicide-related or mental health consultation-related internet use increased suicidal ideation scores only by 0.37–0.55 points during a 6-week observed period. The observation was over a relatively short period. Also, as we have presented in the backgrounds of the group with suicide-related or mental health consultation-related internet use, they have a high probability of being diagnosed with mental disorders associated with suicide, as indicated by the mean scores of K6, the screening scale for detecting CIDI/DSM-IV mood and anxiety disorders. A previous study showed that increasing suicidal ideation was significantly associated with a diagnosis of a principal mood disorder, a diagnosis of a personality disorder and previous suicide attempts [35]. Under such circumstances, those with suicide-related or mental health consultation-related internet use may show more serious consequences such as self-harm, repeated suicidal behaviours and completed suicide in the long term.

### Strengths and limitations

Our internet survey was the first large-scale prospective longitudinal study with a control group chosen by stratified random sampling. There is almost no prospective research in the related area [36]. Most of the previous studies dealing with the effects of suicide-related internet use were based on internet suicide pacts or used a cross-sectional study design [12] [16] [17]. The sampling process in this study was based on the demographic composition of the latest census to minimize sampling and representative bias. In addition, our research clarified the level of suicidal risk of the group with suicide-related or mental health consultation-related internet use, which has not been previously examined.

There were, however, some limitations to our study. First, an online panel survey has some biases such as coverage bias [21]. Second, the T0 participation rate in the survey was relatively low (14.5%). However, the proportion of participation was nearly equal to that of other internet research [21] and the population characteristics of this study were also similar to the latest census data. Third, we showed only short-term results. The long-term effects of suicide-related or mental health consultation-related internet usage are not clear. Fourth, we did not examine the effects of professional mental health services online. This is because there are not yet such validated services in Japan. Fifth, we did not define the contents of the websites or the services rendered in consequence of disclosing suicidal ideation or in seeking mental health consultation with anonymous others. Finally, it is impossible to know which websites participants actually visited and whether the websites they visited were topically related to suicide or not from our research. As an alternative, we asked about the actions that suicidal internet users frequently perform instead of identifying the websites that the participants actually visited in the questionnaire. Because a website may have a function of both prevention and promotion of suicide [37], it is not possible to verify whether the internet use of such a specific website enhances suicide prevention or not. It is useful for us to identify which actions may predict increases or decreases in the internet user's suicidality in order to utilize online helping resources. Therefore the four questions about suicide-related internet usage were made by reference to our previous study about the internet and suicide. However, these items were not used frequently and did not cover all the actions performed on websites that might be suicide-related.

## Conclusion

Increased suicidal ideation was observed in the young and middle-aged with suicide-related or mental health consultation-related internet use. Those who disclosed their suicidal ideation up until a month ago, disclosed their suicidal ideation within the previous month, and browsed for information about suicide methods within the previous month had significantly increased suicidal ideation 7 to 8 weeks later. Meanwhile, mental health consultation via the internet was not useful but may have worsened depression/anxiety. Therefore, regulation of information regarding suicide on the internet should be promoted as a health policy. As a next challenge, new and more effective interventions for vulnerable populations with suicide-related internet use should be developed.
